# Some Unusual Phenomena Attending Anæsthesia

**Published:** 1869-05

**Authors:** Frederic D. Lente

**Affiliations:** Cold Spring, New York.


### ARTICLE X.

### *Some Unusual Phenomena attending Anæsthesia.*

By Frederic D. Lente, M.D., of Cold Spring, New York.

In a recent number of the *Richmond and Louisville Medical Journal*, and copied into the *Boston Medical and Surgical Journal*, is an article by Dr. W. H. Shepherd, entitled "Apparent Exercise of Volition during Anæsthesia complete in all other Respects." The writer thinks it very extraordinary that, although the anæsthesia was apparently perfect, the patient's jaws were firmly closed. "The resistance," he says, "was not such as we find in tonic spasm, but appeared to be the result of voluntary effort, and never yielded, although the anæsthetic (chloroform) was used until the condition of the pupils forbade its further employment." There was, he adds, the usual relaxation of the other muscles of the body.

A rigid condition of certain muscles, under perfect anæsthesia, is not, I think, so very uncommon, and even the perfect exercise of volition is also quite possible, as I have seen exemplified in two cases so remarkable that I am tempted by reading the report of Dr. Shepherd's case, to give them publicity, although the long period which has elapsed since their occurrence, and the possession of no notes, must render the report very meagre.

During the first year of the use of anæsthesia in the New York Hospital, to which I was, at the time, temporarily attached, as surgical assistant, a case of perineal section occurred in the practice, I think, of Dr. Gurdon Buck. The operation was without a guide through the stricture, and the most tedious and difficult of the kind that I have ever witnessed. The patient was on the table over an hour, and the exemption from pain was, all the while, complete ; and yet he was laughing and talking, and making droll remarks, in conversation with the bystanders, most of the time. I remember one of the house staff making notes of some of his queer sayings. The other case was a tedious operation for necrosis of the tibia. The subject was an unusually stupid boy, some fifteen years of age; and yet, under perfect exemption from pain (etherization), he sang numerous comic songs, and made rather witty remarks on the peculiarities of the surgeons around him. The pain, on examining the disease with the probe, previous to the operation, was unusually severe, judging from the outcries of the patient. I cannot remember how often a reapplication of the ether was required, but the sponge was away from the face a good part of the time consumed in the operation. These cases were not deemed extraordinary at the time, and no note was taken of them, as it was then the infancy of anæsthesia, and it was considered quite likely that these events would become not unusual occurrences. But I have never since, to my recollection, met with a record of any thing precisely similar, although it is sufficiently common for patients to recover their mental faculties to such a degree as to enable them to answer questions intelligently, and to cooperate, to some extent, in any necessary movement of the body after the operation has been completed some minutes, and yet feel no pain during the handling and dressing of the wound.

Two cases of troublesome rigidity of muscles, which I call to mind as having happened in my private practice, are briefly as follows: Mrs. N., a rather nervous married lady, about thirty years of age, had visited a neighboring village for the purpose of having a number of teeth extracted; and, according to her statement, had exhausted a pint of ether in the vain attempt of a physician to anæsthetize her. She was assured that such a thing was impossible in her case. However, I undertook the job, and with three ounces of ether, and within the space of four or five minutes (ether enough and time enough to etherize any patient) I had her breathing stertorously, and thoroughly relaxed except the muscles which it was most important to have in that condition, namely, the masseters. It required a strong leverage with a pair of stout forceps to force the jaws open, so that Mr. Davis the dentist in attendance, could extract the teeth. On recovery she exhibited no unusual phenomena, and insisted, for some time, that it was her sister, standing by, and not herself who had undergone the operation.

A young man, and healthy, who had suffered amputation of the leg below the knee some months previously, wished a peg-leg, but the knee-joint was anchylosed in the straight position, and required to be flexed for this purpose. I accordingly administered ether to relax the muscles, as well as to annul pain. The anæsthesia was quite complete, the respiration stertorous, and yet the muscles of the thigh were as rigid as iron. The etherization was pushed to the verge of danger, in the hope of producing relaxation, and thus maintained for several minutes, but unsuccessfully. The patient was therefore allowed to recover somewhat from its effect; and before the return of consciousness or sensibility, the muscles then becoming slightly relaxed, the stump was suddenly flexed by a powerful effort on my part, and the adhesions thus ruptured. In this case, I cannot call to mind whether the other muscles of the body were likewise rigid. In fact, the untoward and perplexing effect of the anæthetic was so annoying that I paid no particular attention to the state of the general muscular system. I cannot pretend to say whether voluntary effort had any influence in determining the muscular contraction in these two cases. A *moderately* firm contraction of the maxillary muscles is not an uncommon occurrence in dental operations under an anæsthetic.—*New York Med Journal.*

